# Why legalizing prostitution in Thailand can help Bangkok regulate commercial sex and curb sex-trafficking systematically and institutionally

**DOI:** 10.3389/fsoc.2023.1227247

**Published:** 2023-11-06

**Authors:** Jason Hung

**Affiliations:** Department of Sociology, University of Cambridge, Cambridge, United Kingdom

**Keywords:** sex trafficking, child trafficking, prostitution, commercial sex, Thailand, policy, safety, labor rights

## Background

Per the United Nations Office on Drugs and Crime's *Global Report on Trafficking in Persons*, sexual exploitation (79%) is known as the most common form of human trafficking at the global level. Predominantly, women and girls are victimized under the presence of human trafficking. Sex trafficking victims refer to those being forced, coerced or placed under the undue influence to participate in prostitution (Roujanavong, 2012). U.S. Department of State (2022)'s *2022 Trafficking in Persons Report* classified Thailand in Tier 2, meaning Bangkok failed to fully meet the minimum standards for the eradication of any trafficking-related activities, despite making significant endeavors correspondingly. Bangkok, per the Report, raised the anti-trafficking capacity, grew the number of trafficking investigations and sentenced officials who were involved in trafficking activities to terms of imprisonment in 2022. In spite of these efforts, Bangkok's number of trafficking prosecutions and convictions in 2022 failed to fall, relative to those in 2021. Thai law enforcement authorities, in addition, purportedly applied inconsistent and ineffective interviewing approaches amid (sex) labor inspection, rendering a raft of trafficking victims to be unidentified (U.S. Department of State, 2022).

Bangkok tightened its legal action against human trafficking by passing the *Prevention and Suppression of Human Trafficking Act* (B.E. 2551) in 2008 (Pink, 2013). However, since then, Thailand has remained placed on the Tier 2 watch list per the *Trafficking in Persons Report*. Such a circumstance hints that solely raising the punitive terms against human trafficking has little to no positive impact on combating any form of trafficking activities domestically. Thailand, as the regionally largest sex tourism destination in Southeast Asia, has a value of USD 6.4 billion in annual revenue generated from underground prostitution activities (Wadekar, 2023). The lucrative sex tourism economy and Bangkok's over-reliance on sex work-related revenues to support its formal and informal economic development have barred Bangkok from effectively curbing sex trafficking activities. While prostitution is deemed illegal, Bangkok has long been known for tolerating commercial sex, especially in tourist-popular locations such as Pattaya, Bangkok and Phuket (McGeough and the Anti-Human Trafficking Cell of Mercyhurst University, 2022; Peter, 2023; Wadekar, 2023). Therefore, law enforcement authorities, when exercising anti-prostitution raids, often turn a blind eye to commercial sex activities, especially when they receive bribes from conventional sex establishment owners or prostitutes themselves (Paramanand, 2019).

## Essay aims

In March 2023, Bangkok drafted a bill to legalize sex work, proposing to allow individuals aged 20 or older to voluntarily enter the sex industry (Charoensuthipan, 2023). While Thai lawmakers have yet to know when the drafted bill will possibly be passed, such a legal endeavor is deemed a remarkable law-making output that helps protect women and girls from facing sexual marginalization and exploitation and is conducive to Bangkok's implementation of more effective and consistent anti-human trafficking operations in the long term. This opinion rationalizes how, if the drafted bill is passed, the legalization of prostitution in Thailand can help domestically curb sex trafficking activities. The opinion will also include some forms of comparative analysis in order to use neighboring countries as examples to justify how the criminalization of sex work has severely marginalized the safety, rights, health and wellbeing of prostitutes, leading commercial sex workers and sex trafficking victims to become systematically and institutionally more traumatized.

## The legalization of sex work and anti-sex trafficking

As a *status quo*, sex workers have been subject to constant physical, verbal, sexual and financial abuse and exploitation by their clients and/or managers (Peter, 2023). However, with their occupation being criminalized, they enjoy no legal rights to seek help from law enforcement and justice departments. They cannot publicly admit that they encounter any form of abuse and exploitation during their engagement in commercial sex, otherwise not only are not unlikely to be legally protected by existing laws but they may plausibly be criminalized.

Also, so long as prostitution becomes legalized, sex workers and victims of sex trafficking at large do not have to pay bribes to corrupt law enforcement authorities during any anti-prostitution raids (Peter, 2023). In recent decades, while Bangkok has been legislatively endeavoring to curb prostitution and trafficking activities, however, such efforts have failed to translate into desirable societal outcomes as law enforcement and justice authorities have been popularly corrupt. Despite the presence of anti-prostitution and -trafficking laws, police and other justice authorities have been keen on accepting bribes from sex trafficking victims and sex workers to turn a blind eye to their engagement in commercial sex. Similar situations have occurred continually in the Philippines. Ample Philippine police officers have the disposition to use anti-trafficking as a cover to extort bribes from prostitutes, commercial sex clients and owners and/or managers of conventional sex establishments. A raft of fake anti-prostitution and anti-trafficking raids were held so as to allow law enforcement authorities to financially exploit the interests of those engaging in commercial sex further and continually (Paramanand, 2019). The societal outcomes presented in Southeast Asia at large in recent decades have demonstrated that prostitution and sex trafficking have largely been tolerated, in part, owing to the loose law enforcement loopholes, despite the criminalization *per se*.

As prostitution is, in theory, criminalized, many conventional sex establishments have been operated in the underground economy. On the one hand, such a circumstance means commercial sex is under- or de-regulated, barring law enforcement authorities from identifying any existing crimes or violence against the interest and safety of sex workers. On the other hand, while, as mentioned, the Thai sex industry earns billions of USD in revenue per year, Bangkok fails to collect tax directly from the activities of commercial sex. So long as commercial sex is legalized, not only can Bangkok gain lucratively from taxing legal industries that benefit from the prevalence of sex tourism, but Bangkok can also earn substantial tax revenues directly from the sex industry. With a continual rise in financial capacity, Bangkok can capitalize on the tax revenue gains on addressing the socioeconomic root causes of prostitution and sex trafficking, including extreme rural poverty, under- or unemployment and educational exclusion against some marginalized and disadvantaged Thai nationals.

Legalizing prostitution does not implicate that Bangkok tolerates sex work more, as same as criminalizing paid sex has failed to minimize such a form of commercial activity. Legalizing prostitution may, nevertheless, help sustainably protect the rights and safety of prostitutes as now they are able to engage in commercial sex above the ground level. Whenever they experience any form of exploitation or abuse, they have the legal right to seek help from local law enforcement authorities. Police officers lose the bargaining power to solely protect the interests of prostitutes under the condition that bribes could be paid. As a result, prostitutes can avoid being financially exploited by both corrupt law enforcement officers and the institutionally unequal power relations that place them in such a limbo. Moreover, like in Indonesia, despite some provincial and local governments imposing commercial sex criminalization, at the national level, prostitution is decriminalized. All Indonesian sex laborers working in regulated brothels have to undertake mandatory, regular HIV and sexually transmitted disease testing in order to protect the sexual health of those engaging in commercial sex. Decriminalizing prostitution in Thailand allows the country to follow the footsteps of Indonesia to better and clearly regulate the sexual health policies within the sex industry (Global Network of Sex Work Projects, n.d.).

Vietnamese officials denounced that underground activities of commercial sex were overly rampant when prostitution was outlawed in their country. Allowing prostitution to operate above the ground level in certain pre-determined areas has been proven to be effective in containing the expansion of commercial sex services in Vietnam over the most recent decade (City Pass Guide, n.d.). In 2012, Hanoi passed the *Law on Handling Administrative Violations* that decriminalized the selling and purchasing of commercial sex (International Centre for Cultural Studies, 2022). Other activities such as pimping, procuring and being involved in underage commercial sex have remained criminalized (International Centre for Cultural Studies, 2022). Such legal endeavors have resulted in proven successes in Vietnam and should be learnt by Bangkok shall Thailand officially legalize prostitution. In order to maintain the protection of human rights, it is of utmost importance to combat any form of sex trafficking, including child trafficking, in Thailand. Bangkok's proposed law to allow those aged 20 or above to sell sex voluntarily hints that the Government intends to allow better regulation and tolerance of sex work while combating any form of sex trafficking, including child trafficking, activities. So long as Thai nationals or foreign nationals in Thailand are maturely aged, the Thai general public should learn that these individuals have full control of their self-agency even if their behaviors (i.e. selling sex) are challenging the moral values and social norms. It is not because the provision of commercial sex should now be deemed socially and morally acceptable, but, in practice, criminalization of prostitution and building a zero-tolerance of sex work are proven to be ineffective and fruitless. Allowing those meeting a pre-set age requirement to sell their bodies voluntarily while criminalizing any form of sex trafficking activities should, in theory, help legally empower sex workers and keep any challenges against moral values and social norms of society to a minimum level.

When prostitution is outlawed, conventional sex establishments' owners can easily circumnavigate laws. For example, in the Philippines, having sex with a girl under the age of 18 is regarded as rape. The involvement in influencing any girls under the age of 18 to undertake sexual intercourse is deemed sex trafficking. Therefore, Philippine bar managers exploit the legal loopholes and commonly present girls under the age of 18 as entertainers rather than sex workers. A patron paying money to bar managers by taking the girl away from the entertainment establishment is marketised as giving “fines” (Redfem and The Fuller Project, 2019). By exploiting these legal loopholes, while Manila passed the *Anti-Trafficking in Persons Act of 2003*, ample underage girls have remained engaged in commercial sex, without any party assuming the legal consequences of sex trafficking (Paramanand, 2019). Bangkok should learn the lesson from the Philippine example and concentrate primarily on curbing sex trafficking activities while protecting the labor rights and safety of above-age prostitutes. More teenagers deciding to enter the sex industry in order to earn “quick and easy” money to support their own families' subsistence needs may plausibly be encouraged to postpone their entry into prostitution until the legal age requirement is satisfied. Moreover, without assuming the responsibilities of arresting and sanctioning above-age prostitutes, relevant Thai law enforcement departments, who are, as a *status quo*, often subject to understaffed and underfunded challenges, can now have sufficient capacity to focus on combating anti-trafficking, including anti-child sex trafficking, activities (Thai PBS World, 2023). Law enforcement endeavors on containing illegal sex work shall become more feasible and effective.

Under the *1996 Prevention and Suppression of Prostitution Act* enacted by Bangkok, prostitution is prohibited. Any person soliciting sex could be fined. Pimps could be fined and imprisoned for up to 10 years. Commercial sex clients having intercourse with children under the age of 15 could be fined and spend up to 6 years in prison. Those having sex with children aged between 15 and 18 could be fined and sentenced to imprisonment for no more than 3 years (Reyes, 2015). These punitive terms should all remain, or even tighten, except for the legalization of engagement in commercial sex once the prostitutes reach the age of 20. In the long term, such a legislative intervention should allow paid sex to take place above the ground level while heavily sanctioning any party who is involved in sex trafficking.

## Conclusions

Ideally passing the proposed law of legalizing prostitution should help Thailand achieve better societal outcomes. However, even if such a bill is not passed, Thai lawmakers should be urged to decriminalize prostitution. Only by destigmatising prostitutes from being labeled as criminals, can sex workers safeguard their labor rights, health and wellbeing when needed. Also, decriminalization or legalization of prostitution, as said, will enable the underresourced and understaffed law enforcement authorities to enjoy an adequate capacity to combat sex trafficking activities, minimizing the risks and odds of anyone being involved in paid sex without their own legal consent.

Decriminalization or legalization of prostitution, by no means, hints at Bangkok's decision to loosen its commercial sex regulations. Such a legal intervention, however, should facilitate the justice system to better systematically and institutionally contain any unlawful act of paid sex and ensure that whoever participating in sex trafficking is severely sanctioned.

Ostensibly the proposed law does not cover the benefits and safety of those under the age of 20. Yet, from an ethical, moral and religious perspective, there is no supporting reason to allow socially disadvantaged Thai women and girls at a premature age to legally engage in commercial sex. Those who are underage, especially, are defined as sex trafficking victims if they take part in prostitution, regardless of whether they enter the sex industry coercively or voluntarily. Sex trafficking remains severely legally sanctioned within and beyond Thailand in order to protect the safety and health of premature girls, especially those of socially disadvantaged origins. Proposing the legal age for prostitution at 20 years old enables those who are lacking life opportunities and financial resources to consider if they would like to enter the sex industry non-coercively. The proposed law, simultaneously, prohibits underage girls to engage in commercial sex so as to avoid them from being subject to child sexual abuse and exploitation. Ultimately, if this proposed law is enacted, those who meet the age requirement and are already in, or considering to enter, the prostitution can enjoy more legal protection within the industry.

## Author contributions

The author confirms being the sole contributor of this work and has approved it for publication.
